# Dimensional analysis yields the general second-order differential equation underlying many natural phenomena: the mathematical properties of a phenomenon’s data plot then specify a unique differential equation for it

**DOI:** 10.1186/1742-4682-11-38

**Published:** 2014-08-27

**Authors:** Gordon R Kepner

**Affiliations:** 1 Membrane Studies Project, PO Box 14180, Minneapolis, MN 55414, USA

## Abstract

**Background:**

This study uses dimensional analysis to derive the general second-order differential equation that underlies numerous physical and natural phenomena described by common mathematical functions. It eschews assumptions about empirical constants and mechanisms. It relies only on the data plot’s mathematical properties to provide the conditions and constraints needed to specify a second-order differential equation that is free of empirical constants for each phenomenon.

**Results:**

A practical example of each function is analyzed using the general form of the underlying differential equation and the observable unique mathematical properties of each data plot, including boundary conditions. This yields a differential equation that describes the relationship among the physical variables governing the phenomenon’s behavior. Complex phenomena such as the Standard Normal Distribution, the Logistic Growth Function, and Hill Ligand binding, which are characterized by data plots of distinctly different sigmoidal character, are readily analyzed by this approach.

**Conclusions:**

It provides an alternative, simple, unifying basis for analyzing each of these varied phenomena from a common perspective that ties them together and offers new insights into the appropriate empirical constants for describing each phenomenon.

## Background

What is the nature of the mathematical commonality that underlies each of the following empirically established equations that describe, collectively, numerous and diverse phenomena: $a\cdot e^{bt};\;a\cdot t\;/\left(b+t\right);\;a\cdot t^{b}.;\;a+b\cdot t^{2};\;a+b\cdot \ln t;\;a\cdot e^{b\cdot t^{2}/2}$ and others, such as the Hill equation for multi-site ligand binding? Each of these functions contains two non-zero empirical constants with physical units, excepting (a · *t*^b^) with one empirical constant and one numerical coefficient as exponent. A second-order differential equation (D.E.) free of these parameters can describe each, using only the variables [*t, y, dy / dt, d*^2^*y / dt*^2^].

It is essential that any D.E. describing a natural phenomenon reconcile the units of the terms on both sides of the D.E. The restriction places stringent requirements on the form of the D.E. This is particularly so when the D.E. is also required to be free of empirical constants. Thus, it focuses on the functional relationship of the variables that govern the phenomenon’s behavior, free of empirical constants.

The analysis uses the general underlying D.E., based on a dimensional analysis of the physical variables, together with the directly observable mathematical properties of the experimental data plot unique to the phenomenon. This yields a specific second-order D.E. that underlies the mathematical function describing each phenomenon’s data plot.

The approach is new, unifying, and simple (Occam’s razor). Its restrictive features reflect the essential requirements of dimensional analysis. Integrating yields the true empirical constants for the mathematical function as defined by the boundary conditions that uniquely describe the data plot. Practical examples of natural phenomena are analyzed to derive a specific D.E. and the unique solution function that describes the phenomenon’s behavior for the given boundary conditions.

This analysis technique departs significantly from others that may start with assumptions about the phenomenon’s mechanism, its variables, and empirical constants. Typically, these lead to either an algebraic function with its two empirical constants already assumed or a first-order D.E. with its one assumed empirical constant. Suppose there was a phenomenon of interest but no obvious mechanism on which to base a derivation of the function describing the data plot. How then would it be analyzed mathematically? This can be done in a systematic way using only the general D.E. and the data plot’s mathematical properties. Thus, a mechanism is not an *a priori* condition for undertaking the analysis or for describing mathematically the data plot generated by Nature. It is irrelevant to this mathematical analysis.

In studying a mechanism *per se*, a necessary requirement is that it be able to derive the mathematical function describing the data plot, which has previously been derived in some independent manner. This still does not establish definitively the proposed mechanism as the correct one, nor the correctness of its assumed empirical constants. The empirical constants that emerge in such an approach may not be the same as those that emerge from integrating the second-order D.E. with its specified boundary conditions. This is demonstrated in some of the examples presented here.

Each approach provides different information about the phenomenon. The D.E. approach is only concerned with the fact of the data plot and its role in specifying a D.E. that leads to the algebraic function, with its empirical constants, that describes the data plot. The other approach assumes a mechanism and tests it against the already accepted algebraic function for the data plot. They are not incompatible. It is not an *a priori* requirement that the only interesting analysis of a phenomenon’s data plot must start with a proposed mechanism. The D.E. approach presents new challenges to think about mechanism in a different way in order to derive the D.E. This paper does not also attempt to do this for every example presented.

## Mathematical method

The analysis requires the following:

the experimental data plot for a given natural phenomenon.

the assumption that a function with two non-zero parameters, such as empirical constants having physical units or numerical exponents, describes a particular phenomenon’s data plot.

Seek a general second-order D.E. based on physically reasonable assumptions about the properties of the four variables [*t, y, dy / dt, d*^2^*y / dt*^2^] and the rational restrictions they place on this D.E. Apply the principles of dimensional analysis, which mandate that the units on each side (LHS, RHS) of the D.E. be identical. This restriction sorts out the units requirement for each possible term involving some combination of the physical variables that could fit into the D.E., on the RHS — the LHS is always the second derivative of the independent variable. Establishing the relationship of *d*^2^*y / dt*^2^ to the other variables [*t, y, dy / dt*] yields the second-order D.E. that regulates acceleration. This regulatory process resides in the specific interplay of the terms containing these variables.

Seek to derive the form taken by the function of the variables as linear combinations of terms containing those variables, which meet the assumptions to produce the general D.E. Then choose examples of well-known natural phenomena. Use the general D.E. and the mathematical properties of the phenomenon’s experimental data plot to derive a D.E. that integrates to give the function describing the data plot. Evaluate the empirical constants arising from the integrations using the boundary conditions on the data plot.

## Results

### Deriving general D.E

Assume the second derivative, *d*^2^*y* / *dt*^2^ (the acceleration), depends on the variables (*t, y*) and the velocity (*dy* / *dt*). Assume the D.E. has the restricted general form

(Ia) $$\frac{d^{2}y}{dt^{2}}=f\left[t,y,\left(\frac{\mathrm{dy}}{\mathrm{dt}}\right)\right]$$

where *f* [] meets the following conditions:

1. excludes empirical constants.

2. excludes non-algebraic terms.

3. excludes *d*^*2*^*y / dt*^*2*^ identically zero.

Assume *f* [] is a linear combination of variables taking the form, *t*^p^ · *y*^q^ · (*dy / dt*)^r^. Consider the integer values (+2, +1, 0, -1, -2) for the powers (p, q, r) on these variables. This gives three variables, *t*^p^ · *y*^q^ · (*dy / dt*)^r^, call these n = 3. Each can exist in five different states (+2, +1, 0, -1, -2), call these m = 5. Thus, when taken three at a time, there are m^n^ = 5^3^ = 125 possible combinations, called terms.

These natural phenomena must have variables with measurable units. Apply dimensional analysis to both sides of the D.E. Thus, the LHS units are (Y / T^2^), which means that *f* [*t*, *y*, *dy / dt*] must have units of (Y / T^2^). This requirement reduces the 125 possibilities to just three: (1 / *t*) · (*dy / dt*), (1/ *y*) · (*dy / dt*)^2^, and *y / t*^2^ that meet the units requirement for the RHS. Non-algebraic terms such as (sin *t*) or (ln *y*) are excluded from this general second-order D.E. because of the units requirements (no non-zero empirical constants with physical units, such as in sin ß*t* or ln k*t*). The general expression for the terms that satisfy the units requirement is

(Ib) $$m_{r}\cdot {\left(\frac{\mathrm{dy}}{\mathrm{dt}}\right)}^{r}\cdot \frac{t^{\left(r-2\right)}}{y^{\left(r-1\right)}}=m_{0}\cdot {\left(\frac{\mathrm{dy}}{\mathrm{dt}}\right)}^{0}\cdot \frac{y}{t^{2}}+m_{1}\cdot {\left(\frac{\mathrm{dy}}{\mathrm{dt}}\right)}^{1}\cdot \frac{1}{t}+m_{2}\cdot {\left(\frac{\mathrm{dy}}{\mathrm{dt}}\right)}^{2}\cdot \frac{1}{y}$$

where m_r_ ≥ 0 is the integer numerical coefficient for the r_th_ term.

Experience and physical intuition support the assumption that *y / t*^2^, either alone or in combination with other terms does not lead to a function describing common natural phenomena. When integrated, none of these 16 combinations gives a D.E. with recognizable solution applicable to any common natural phenomenon that is described by two parameters. So, it is excluded. Therefore, m_0_ = 0 here. As the results will show, none of the common phenomena analyzed here require the *y / t*^2^ term.

Thus, this analysis focuses initially on m_1_ and m_2_ equal to 0 or 1. The other two terms can be combined to give linear combinations. This yields eight distinct cases for the RHS, where each case can integrate to a variety of different functions, depending on the values of m_r_ and the boundary conditions.

Thus, for m_1_ = 1 = m_2_.

a. (*dy* / *dt*) · [(1 /*t*) + (1 /*y*) · (*dy* / *dt*)] = [(1 /*t*) · (*dy* / *dt*)^1^] + [(1 /*y*) · (*dy* / *dt*)^2^]

b. (*dy* / *dt*) · [(−1 /*t*) – (1 /*y*) · (*dy* / *dt*)]

c. (*dy* / *dt*) · [(1 /*t*) – (1 /*y*) · (*dy* / *dt*)]

d. (*dy* / *dt*) · [(−1 /*t*) + (1 /*y*) · (*dy* / *dt*)]

For m_1_ = 1, and m_2_ = 0.

e. (*dy* / *dt*) · [(1 /*t*)]

f. (*dy* / *dt*) · [(−1 /*t*)]

For m_1_ = 0, and m_2_ = 1.

g. (*dy* / *dt*) · [(1 /*y*) · (*dy* / *dt*)]

h. (*dy* / *dt*) · [(−1 /*y*) · (*dy* / *dt*)]

Allowing other values for m_r_ greatly increases the number of possible linear combinations. Some examples will be presented. In theory, m_r_ could take a fractional value.

Consider one example of a linear combination for the second-order D.E., case **a**.

(Ic) $$\frac{d^{2}y}{dt^{2}}=\frac{\mathrm{dy}}{\mathrm{dt}}\cdot \left[\frac{1}{t}+\left(\frac{1}{y}\right)\cdot \left(\frac{\mathrm{dy}}{\mathrm{dt}}\right)\right]$$

which can be rearranged to

(Id) $$\left(\frac{d^{2}y}{dt^{2}}\right)\cdot \mathrm{dt}=d\left(\frac{\mathrm{dy}}{\mathrm{dt}}\right)=\left(\frac{\mathrm{dy}}{y}\right)\cdot \left[\left(\frac{y}{t}\right)+\left(\frac{\mathrm{dy}}{\mathrm{dt}}\right)\right]$$

This reveals the key relationship between the slope, (*dy / dt*), and the coordinates slope, (*y / t*). The relationship is evaluated directly from the data plot. It is characteristic for each phenomenon and modulates the behavior of the fractional change in the dependent variable, (*dy / y*). It governs the LHS, the acceleration/deceleration.

The task is to establish the specific linear combination that underlies the data plot of a particular natural phenomenon with its boundary conditions. For the same values of m_r_, any of these D.E.s can describe more than one phenomenon’s data plot when there are different boundary conditions present. Then each function is a unique solution of the D.E. plus boundary conditions.

Each data plot exhibits mathematical properties and boundary conditions that, taken together with these established cases for *f* [], leads to an *f* [] that mathematically describes the specific data plot. These properties include:

1. The sign of *d*^2^*y* / *dt*^2^ is obtained directly from the tangent to the experimental data plot of *y* versus *t* for each phenomenon. The sign of *dy / dt* is also obtainable from direct observation of the plot.

2. The behavior and boundedness of *d*^2^*y* / *dt*^2^ when *t* goes to zero or to infinity.

3. The behavior and boundedness of *dy / dt* when *t* goes to zero or to infinity.

4. The relative magnitudes of the slope, *dy / dt*, and coordinates slope, *y / t*.

The terms inside the [] of equation (Ic) can be compared to determine if: (*y / t*) < (*dy / dt*) or > (*dy / dt*). Given (*y / t*), the coordinates slope for a line drawn from the origin to a point on the data plot, and (*dy / dt*), the slope at that point, it is possible to establish whether (*dy / y*) is > or < (*dt / t*). For example, if (*dy / dt*) < (*y / t*), then (*dy / y*) < (*dt / t*).

This is applied in another useful form of the D.E., which is directly integrable, the fractional change form, shown below.

(Ie) $$\left(\frac{\frac{d^{2}y}{dt^{2}}\cdot \mathrm{dt}}{\frac{\mathrm{dy}}{\mathrm{dt}}}\right)=\frac{\mathrm{dt}}{t}+\frac{\mathrm{dy}}{y}$$

Specific useful tests to rule out the incorrect combinations include:

1. At a limit, such as when *t* goes to zero, is *dy / dt* or *d*^2^*y* / *dt*^2^ bounded or not? Does the RHS of the D.E. give a bounded or unbounded value?

2. Eliminate each case where the sign of the RHS, (*dy / dt*) · *f* [], is not the same as the LHS, *d*^2^*y* / *dt*^2^.

3. Compare the slope (*dy* / *dt*) to the coordinates slope (*y* / *t*) at a point on the plot, to determine the relative magnitudes of (*dt / t*) and (*dy / y*) — see equation (Id) — or (*y / t*) and (*dy / dt*) — see equation (Ic).

4. Each example provides a D.E. that has a unique solution that describes the particular natural phenomenon’s data plot and its boundary conditions.

5. As examples accrue, so the number of possible cases (**a.** through **h.**) that might apply to the next example must diminish, under the assumption that no two of these eight cases give the same function for the same boundary conditions.

6. All the examples are real phenomena with physical variables. It is assumed that as *t* goes to zero, the *d*^2^*y* / *dt*^2^ does not go to infinity, therefore bounded.

Physically, equation (Ic) defines the way acceleration (deceleration) depends on velocity—modified by a linear combination of [1 / *t*] and [(1 / y) · (*dy / dt*)]. Only these two modifying factors need to be taken into account. This places restrictions on what needs to be considered for any proposed physical relationship or mechanism.

### Examples

#### Radioactive decay

**Figure 1:**
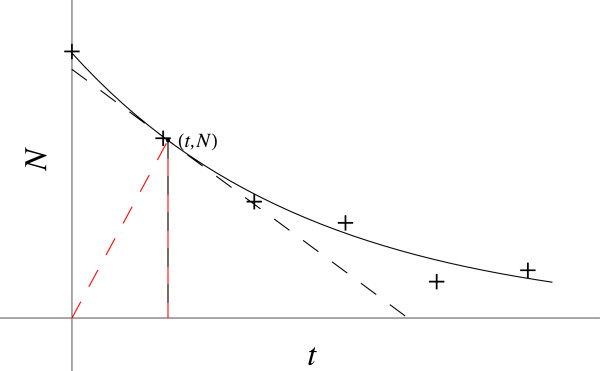
**Radioactive decay.** The red dashed line gives the coordinates slope (*N / t*). The black dashed line gives the slope at this point (*dN / dt*).

Let *N* / N_0_ be the fraction of radioactive atoms remaining at time *t*. The *N* versus *t* plot (Figure 1) has its tangent below the curve, so the LHS of the D.E. must be positive and therefore the RHS also.

The slope, *dN / dt*, is negative and decreasing in magnitude as *t* increases. As *N* has a definite value at the origin, N_0_, it is not zero. It is expected that (*d*^2^*N / dt*^2^) and *dN / dt* stay bounded as *t* goes to zero (as does 1 / *N*), whereas the term (1 / *t*) goes to infinity. The coordinates slope, *N / t*, is greater than the slope, *dN / dt*.

Cases **a.**, **c.**, **e.** and **h.** give a negative RHS, incorrect. Cases **b.**, **d.** and **f.** give a RHS that goes to infinity as *t* goes to zero, whereas the LHS is bounded. Thus, case **g.**, which is bounded on the RHS, gives

(1a) $$\frac{d^{2}N}{dt^{2}}=\frac{\mathrm{dN}}{\mathrm{dt}}\cdot \left[\left(\frac{1}{N}\right)\cdot \left(\frac{\mathrm{dN}}{\mathrm{dt}}\right)\right]$$

Therefore, after rearranging, the second-order D.E., free of empirical constants, is

(1b) $$\frac{\frac{d^{2}N}{dt^{2}}\cdot \mathrm{dt}}{\frac{\mathrm{dN}}{\mathrm{dt}}}=\frac{\mathrm{dN}}{N}$$

so

(1c) $$\ln|\frac{\mathrm{dN}}{\mathrm{dt}}|=\ln N+C$$

and

(1d) $$\frac{\mathrm{dN}}{\mathrm{dt}}=\pm e^{C}\cdot N=C_{1}\cdot N$$

giving

(1e) $$N=C_{2}\cdot e^{C_{1}\cdot t}$$

The empirical constants, C_1_ and C_2_, evaluated for this case, are C_2_ = N_0_ and C_1_ = - k, which is the decay constant for the radioactive atom. The natures of C_1_ and C_2_ are particular to each phenomenon. For the phenomenon of unrestrained exponential population growth, C_1_ = + k, the growth rate, and C_2_ is the starting population.

The LHS of equation (1a), deceleration, is the differential change in *dN / dt*. The RHS is the velocity of decay modulated by [(1 / *N*) · (*dN / dt*)]. The assumption of the inherent random nature of radioactive decay means the fractional change in *N,* (*dN / dN*), per increment, *dt*, at a given *t* is the same. Thus, [(*dN / N*) / *dt*]_t_ is a constant.

#### Probability distribution

**Figure 2:**
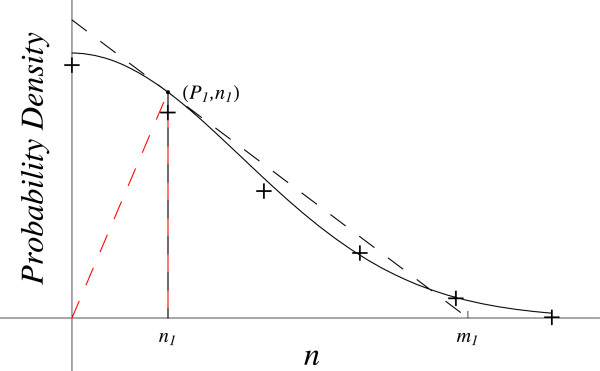
**Probability distribution.** The red dashed line gives the coordinates slope (*P*_*1*_*/ n*_*1*_). The black dashed line gives the slope at this point (*dP / dn*)_1_.

Numerous phenomena involving random processes generate a classic bell-shaped curve, the standard normal distribution (SND), Figure 2.

Let *P* be the probability density of a random event of magnitude *n*. As *n* increases from the origin to the inflection point, the tangent to the curve remains above the line, so *d*^2^*P* / *dn*^2^ and *dP / dn* are negative and also increasing in magnitude. However, from the inflection point to infinity, the tangent is below the curve so *d*^2^*P* / *dn*^2^ is positive and decreasing in magnitude, while *dP / dn* is negative and decreasing in magnitude. From zero to the inflection point, (*dP / dn*) · (1 / *P*) gives the correct sign (negative) for the RHS, but not from the inflection point to infinity where the LHS is now positive. Conversely, from the origin to the inflection point, (*dP / dn*) · [(1 / *P*) · (*dP / dn*)] is positive, which gives the wrong sign. From the inflection point to infinity it gives the correct sign (positive)—because the tangent is below the curve and so LHS is positive, see Figure 2.

Consider the RHS of each of the following linear combinations.

c. (*dP* / *dn*) · [(1/ *n*) – (*dP* / *dn*) · (1 /*P*)], fails because it is always negative and so cannot accommodate the change in sign of *d*^2^*P* / *dn*^2^ as it passes through the inflection point.

d. [(*dP* / *dn*) · [(− 1/ *n*) + (*dP* / *dn*) · (1 /*P*)], fails because it is always positive.

b. (*dP* / *dn*) · [− (1/ *n*) – (*dP* / *dn*) · (1 /*P*)] = (*dP* / *dn*) · *P* · [− (*P* / *n*) – (*dP* / *dn*)]. As *n* approaches zero, (*P / n*)_0_ > magnitude of (*dP / dn*)_0_ because (*dP / dn*)_0_ approaches zero, while (*P*_0_ / *n*)_0_ goes to infinity. Therefore, (*dP / dn*) · (- *P / n*) is positive and the larger term. Thus, case **b.** fails because the RHS has to be negative here (tangent above the curve).

a. (*dP* / *dn*) · [(1/ *n*) + (*dP* / *dn*) · (1 /*P*)] = (*dP* / *dn*) · *P* · [(*P* / *n*) + (*dP* / *dn*)].

This gives a negative RHS as required, because (*P / n*) is greater than the magnitude of (*dP / dn*).

Further, (*P / n*) continues to decrease as *n* increases to infinity, whereas the magnitude of (*dP / dn*) increases until it reaches a maximum at the inflection point and then steadily decreases. The sign of *d*^2^*P* / *dn*^2^ changes from negative to positive at the inflection point, because the tangent is below the curve past the inflection point. After that the magnitude of the negative *dP / dn* becomes greater than (*P / n*), so (*dP / dn*) · *P* · [+ (*P / n*) + (*dP / dn*)] then goes positive once the tangent is below the curve. It follows that (*P / n*)_infl_ = (*dP / dn*)_infl_ in order for this transition in sign to occur. Thus, case **a.** gives,

(2a) $$\frac{\left(\frac{d^{2}P}{dn^{2}}\right)\cdot \mathrm{dn}}{\left(\frac{\mathrm{dP}}{\mathrm{dn}}\right)}=\frac{\mathrm{dP}}{P}+\frac{\mathrm{dn}}{n}$$

and

(2b) $$\frac{\mathrm{dP}}{\mathrm{dn}}=C_{1}\cdot \left(P\cdot n\right)$$

giving

(2c) $$P=C_{2}\cdot e^{C_{1}\cdot n^{2}/2}$$

For C_1_ = -1, with units of (1 / *n*^2^), and C_2_ = P_0_ = 1/√2π = 0.399, with units of probability density, this becomes the probability density function for the (SND), with mean = 0, and standard deviation = 1. The RHS of equation (2a) is thus the unique linear combination of the variables that yields the *P* versus *n* plot for the (SND).

One intuitive approach to deriving the SND, based on the fractional change concept, looks at the relationship between *n* and the fractional change in the probability velocity, where *p* = ∫ *P* · *dn*. Define Δ_f_ (*dp* / *dn*) = [(*d*^2^*p* / *dn*^2^) · *dn*] / (*dp* / *dn*).

It seems reasonable, initially and for simplicity, to assume that neither the velocity nor deceleration of the probability depends on the dependent variable, *p*. Therefore, ∆_f_ (*dp / dn*) depends only on the independent variable, *n*.

Using *P* = (*dp / dn*) and *dP / dn* = *d*^2^*p / dn*^2^ allows determination of the sign of ∆_f_ (*dp / dn*) from the slope of the *P* versus *n* data plot in Figure 2 and the relation

(2d) $$\frac{\frac{d^{2}p}{dn^{2}}}{\frac{\mathrm{dp}}{\mathrm{dn}}}=\frac{\left(\mathrm{dP}/\mathrm{dn}\right)}{P}$$

where (*dP / dn*) is always negative, so ∆_f_ (*dp / dn*) is negative.

The possibility that ∆_f_ (*dp / dn*) might depend on [- (1 / *n*)] can be excluded by considering the behavior of [(1 / *P*) · (*dP / dn*)] at small values of *n*. There, (1 / *P*) is at its smallest and (*dP / dn*) is also small, so their product is even smaller. Whereas, (1 / *n*) is at its largest at small *n*. The behavior of [(1 / *P*) · (*dP / dn*)] mirrors the behavior of *n*, as *n* increases.

Let ∆_f_ (*dp / dn*) depend directly on *n*, giving

(2e) $$\Delta_{f}\left(\frac{\mathrm{dp}}{\mathrm{dn}}\right)=\left(\alpha \cdot n\right)\cdot \mathrm{dn}=-n\cdot \mathrm{dn}$$

For the SND, α = -σ = -1, with units of (1 / *n*^2^), then

(2f) $$p=\int \mathrm{dp}=\int C_{2}\cdot e^{‒n^{2}/2}\cdot \mathrm{dn}=\int P\cdot \mathrm{dn}$$

This yields

(2g) $$\frac{d^{2}p}{dn^{2}}=\frac{\mathrm{dP}}{\mathrm{dn}}=-n\cdot P$$

So, at any point on the *P* versus *n* plot, the probability’s deceleration equals the probability density’s slope and that equals the area (- *n · P*), see Figure 2. A plot of (- *n · P*) versus *n* reveals the behavior of the probability’s deceleration and the probability density’s velocity. They steadily decrease to reach a minimum at *n* = 1, then increase passing through an inflection point at *n = √*3 and approach zero as *n* goes to infinity.

The fractional change in the probability density slope, ∆_f_ (*dP / dn*) = [(1 / *n*) – *n*] · *dn*. Together with ∆_f_ (*dp / dn*) = - *n · dn,* these second-order D.E.s define the essential mathematical constraints that govern the continuous SND.

#### Laminar flow in blood vessel

**Figure 3:**
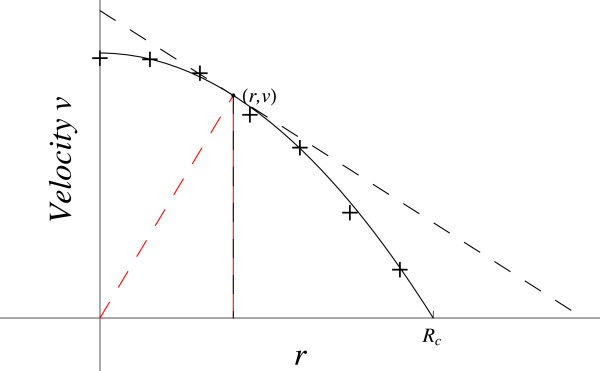
**Laminar flow in blood vessel.** The red dashed line gives the coordinates slope (*v / r*). The black dashed line gives the slope at this point (*dv / dr*).

Consider the commonly used description of the velocity of laminar blood flow through the uniform length of a cylindrical blood vessel, where R_c_ = cylinder radius [1]. The velocity, *v*, is a function of the distance from the center of the vessel, *r*. Experimental data show (Figure 3) that *v* is a parabolic function of *r*, in this simple case, with maximum velocity at the vessel’s center where *r* = 0.

This analysis uses the standard assumptions to simplify the problem, as others have done, and treat it as simple laminar flow in a cylindrical vessel. The value of *v* decreases as *r* increases to *r* = R_c_ at the vessel wall, where *v* = 0, the non-slip condition, and *dv / dr =*0 because of axial symmetry. The tangent is above the curve so *d*^*2*^*v* / *dr*^2^ is negative as is *dv / dr*, and both are bounded as *r* goes to zero. Both are increasing in magnitude as *r* increases to R_c_. The velocity, *v,* is steadily decreasing in magnitude as *r* approaches the vessel wall at radius R_c_.

Cases **b.**, **d.**, **f.** and **g.** give a positive RHS, incorrect. Of the remaining cases, **a.**, **c.** and **h.** contain (1 / *v*) · (*dv / dr*)^2^. When *v* goes to zero, this goes to infinity so the RHS ≠ LHS, which is bounded. The case **e.** does not go to infinity. Additionally, when *r* goes to zero then (*dv / dr*) / *r* goes to (0 · ∞). Applying l’Hopital’s Rule gives (*d*^*2*^*v* / *dr*^2^)_0_ / 1 = LHS.

Therefore,

(3a) $$\frac{d^{2}v}{dr^{2}}=\frac{\mathrm{dv}}{\mathrm{dr}}\cdot \left[\frac{1}{r}\right]$$

so

(3b) $$\frac{\mathrm{dv}}{\mathrm{dr}}=C_{1}\cdot r$$

and

(3c) $$v=C_{1}\cdot \frac{r^{2}}{2}+C_{2}$$

When *r* = 0, then *v* = *v*_max_ = C_2_. When *v* = 0, then *r =* R_c_ and so C_1_ = -2 · *v*_max_ / (R_c_)^2^. This gives

(3d) $$v=\frac{v_{\text{max}}}{R_{c}^{2}}\cdot \left[R_{c}^{2}-r^{2}\right]$$

Standard formulations for the velocity of flow have assumed a constant containing three factors: the pressure drop, ∆P; the fluid viscosity, μ; and the vessel length, L; related by K = ∆P / (4 · μ · L). Therefore, [*v*_max_ / (R_c_)^2^] = ∆P / 4 · μ L. The approach developed here to derive equation (3d) relied on the relationship between the variables, independent of assumptions about empirical constants. It also showed that a complete description of the data plot is obtainable from *v*_max_ and R_c_, well-defined and directly measurable quantities.

The D.E. also emerges from the assumption that the jolt (rate of change of *dv / dr = d*^2^*v / dr*^2^) is directly dependent on the acceleration, *dv / dr*, and is independent of velocity, *v*. It depends only on the geometry as defined by *r.* Therefore, the RHS must have units of *v / r*^2^ and so it must take the form (*dv / dr*) · (1 / *r*), revealing the inverse dependence on *r* as expected.

#### Response to sound intensity

**Figure 4:**
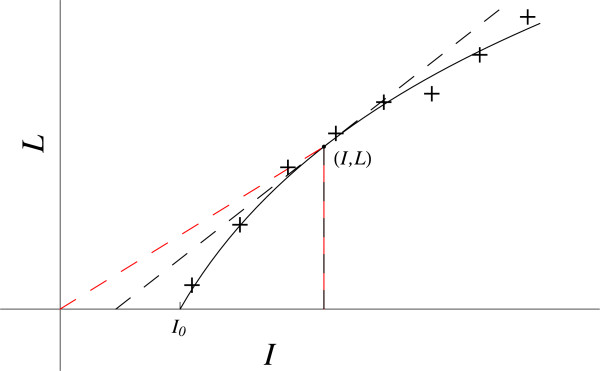
**Response to sound intensity.** The red dashed line gives the coordinates slope (*L / I*). The black dashed line gives the slope at this point (*dL / dI*).

The subjective response of humans to sound intensity can be described mathematically [1]. Let the intensity of sound equal *I*, and the perceived loudness equal *L*. The experimental data plot (Figure 4) has the initial value I_0_ at *L* = 0, and rises steadily with decreasing slope as *I* increases.

The slope, *dL* / *dI*, is positive and *d*^*2*^*L* / *dI*^2^ is negative because the tangent is above the curve. Therefore, both the LHS and RHS must be negative. The LHS, (*d*^2^*L / dI*^2^), is bounded when *L* goes to zero, where *I* = I_0_.

Cases **a.**, **c.**, **e.** and **g.** give a positive RHS, incorrect. Cases **b.**, **d.** and **g.** go to infinity on the RHS when *L* goes to zero. Case **f.** gives a finite RHS, thus

(4a) $$\frac{\left(\frac{d^{2}L}{dI^{2}}\right)\cdot \mathrm{dI}}{\left(\frac{\mathrm{dL}}{\mathrm{dI}}\right)}=-\frac{\mathrm{dI}}{I}$$

and

(4b) $$\ln\left(\frac{\mathrm{dL}}{\mathrm{dI}}\right)=-\ln I+\ln\;C_{1}$$

thus

(4c) $$L=C_{2}+C_{1}\cdot \ln I$$

The lowest intensity that can be heard, the threshold of audibility, is defined as I_0_. When *L* = 0, then *I* = I_0_, giving

(4d) $$L=C_{1}\cdot \ln\left(\frac{I}{I_{0}}\right)$$

The value of C_1_ is determined experimentally and is referenced to a tone of 1000 Hz for humans, for this relationship.

Many phenomena that involve detecting differences in human sensation in response to stimuli have been studied, leading to a general law (Weber’s law) that is a reasonable approximation to reality, within limits on the range of the stimuli [1]. For the example here, assume that detection depends on the increase in a stimulus being a constant percentage of the stimulus. Thus, ∆_f_ (*dL / dI*) depends on ∆_f_ (*I*). Thus, for a negative LHS,

(4e) $$\Delta_{f}\left(\frac{\mathrm{dL}}{\mathrm{dI}}\right)=\frac{\frac{d^{2}L}{dI^{2}}\cdot \mathrm{dI}}{\frac{\mathrm{dL}}{\mathrm{dI}}}=-\Delta_{f}\left(I\right)=-\frac{\mathrm{dI}}{I}$$

as in equation (4a).

#### Power law examples

**Figure 5:**
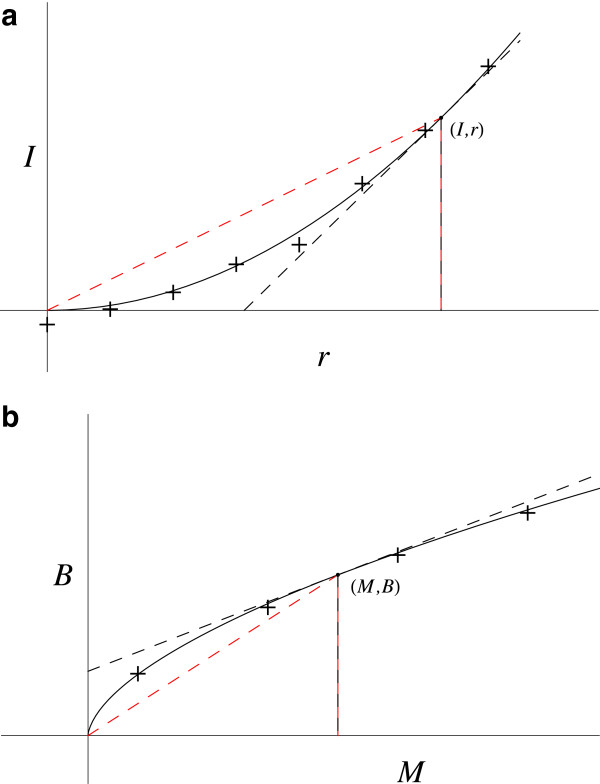
**Two power law functions. a.** Light intensity as a function of iris radius. The red dashed line gives the coordinates slope (*I* / *r*). The black dashed line gives the slope at this point (*dI / dr*). **b.** Basal metabolic rate versus organism mass. The red dashed line gives the coordinates slope (*B / M*). The black dashed line gives the slope at this point (*dB / dM*).

Consider the iris of the eye with radius, *r*. A small change in its diameter alters the intensity, *I*, of entering light [1]. The data plot (Figure 5a) has a tangent below the curve, so the second derivative is positive. Therefore, the RHS of the D.E. must be positive. This eliminates cases that give a negative RHS: **b.**, **c.**, **f.** and **h.** Case **c.** is negative because from the data plot, (*dI / dr*) > *I / r*.

The cases that give a positive RHS are:

a. (*dI* / *dr*) · [(1 /*r*) + (1 /*I*) · (*dI* / *dr*)].

d. (*dI* / *dr*) · [(− 1 /*r*) + (1 /*I*) · (*dI* / *dr*)].

e. (*dI* / *dr*) · (1/ *r*).

g. (*dI* / *dr*) · [(1 /*I*) · (*dI* / *dr*)].

Three cases (**a.**, **e.**, **g.**) have been employed for the first three examples. This leaves case **d.** for this example with its boundary conditions. Additionally, only **d.** satisfies the condition that the RHS equals the bounded LHS as *r* goes to zero, for any bounded value on the LHS (zero or nonzero). Thus,

(5a) $$\frac{d^{2}I}{dr^{2}}=\frac{\mathrm{dI}}{\mathrm{dr}}\cdot \left[\frac{-1}{r}+\left(\frac{1}{I}\right)\cdot \left(\frac{\mathrm{dI}}{\mathrm{dr}}\right)\right]$$

so

(5b) $$C_{1}=\frac{\mathrm{dI}/I}{\mathrm{dr}/r}$$

where C_1_ is the dimensionless numerical scaling factor defined by the ratio of the fractional changes in the variables. It is not an empirical constant, but a numerical coefficient without units. Therefore it is redefined here to C_1_ = n, giving

(5c) $$I=C_{2}\cdot r^{n}$$

One typical approach to analyzing the parameter, n, has been to use a log-log plot of the data, where the slope gives the scaling factor, n. A plot of ln *I* versus ln *r*, when linear, yields n. The plot in Figure 5a applies when n > 1. Intuitively, it is reasonable to assume that ∆_f_ (*dI / dr*) is directly dependent on *I* and inversely dependent on *r.* Thus,

(5d) $$\Delta_{f}\left(\frac{\mathrm{dI}}{\mathrm{dr}}\right)=\frac{\frac{d^{2}I}{dr^{2}}\cdot \mathrm{dr}}{\frac{\mathrm{dI}}{\mathrm{dr}}}=\Delta_{f}\left(\frac{I}{r}\right)=\frac{\mathrm{dI}}{I}-\frac{\mathrm{dr}}{r}$$

as in equation (5a).

Many important natural phenomena can be described when 0 < n < 1. These include a broad class of allometric phenomena that describe how basic and complex natural phenomena scale with size, typically following a power law. The value of n is generally thought to be a multiple of (1 / 4), [2]. The example presented in Figure 5b represents the relation between basal metabolic rate, *B*, and organism mass, *M*. Again, assume ∆_f_ (*dB / dM*) = ∆_f_ (*B / M*) to obtain equation (5a).

Analyzing the four cases that give a negative RHS (**b.**, **d.**, **f.** and **h.)** as was done in the previous example leads, as expected, to case **d.**, giving

(5e) $$\frac{d^{2}B}{dM^{2}}=\frac{\mathrm{dB}}{\mathrm{dM}}\cdot \left[\frac{-1}{M}+\frac{1}{B}\cdot \frac{\mathrm{dB}}{\mathrm{dM}}\right]$$

so

(5f) $$B=C_{2}\cdot M^{n}$$

where 0 < n < 1. The slope of the log-log plot gives n, when linear. Commonly, the value found in such experiments is n ≈ (3 / 4). It has been suggested that C_2_ has biological significance [3]. It is usually treated as a normalization factor. There is an ongoing search for a mechanism to explain this function [2,4]. “The belief that metabolic rate and other physiologic variables are related to body mass by a two-variable power law is assumed *a priori* in FNT (Fractal Network Theory). Yet it is not deducible from any principles of physics, geometry or biology, so it must be considered an unacknowledged *ad hoc* assumption.” [4], see also [5]**.** Thus, as pointed out in the Background, when no mechanism exists then the D.E. and the data plot’s properties provide the mathematical basis for the function that serves as the benchmark test for the relevance of any proposed mechanism and the appropriate empirical constants.

The question of the awkward units for C_2_ and *M*^(3/4)^, (grams)^3/4^, seems not to arise. Suppose a different value of *M* was used, say *M*_c_ that gave a more precise estimate of the actually metabolizing cellular mass. This could yield a relation where *B*_c_ = K_c_ · (*M*_c_)^n^, where n could be an integer. For example, correcting for factors such as fluid in the bladder, waste in the bowel, blood plasma volume, and extracellular fluid would produce a smaller *M*_c_ that might lead to an integer value of n. This could yield a more realistic set of units. If n = 1, then K_c_ = (cal / hr) / g. Thus, K_c_ gives the basal metabolic rate per gram of the presumed metabolizing cellular mass, *M*_c_.

This approach to identifying the metabolizing cell mass could aid the search for a mechanism. The D.E. with its factors modifying the *dB* / *dM* — (1 / *B*) and (1 / *M*) · (*dB / dM*) — offers another approach to developing a mechanism based on the metabolizing cell mass. Such data analysis could produce a value of m > 1, so the plot would take the form of Figure 5a.

#### Multiple ligand binding

[The subsequent equations (6a and 7a) will illustrate the effect of allowing m_r_ to have values greater than one, as well as when m_1_ does not equal m_2_. As will be shown, equation (6a) has m_1_ = 5 and m_2_ = 2; and equation (7a) has m_1_ = 2 = m_2_].

**Figure 6:**
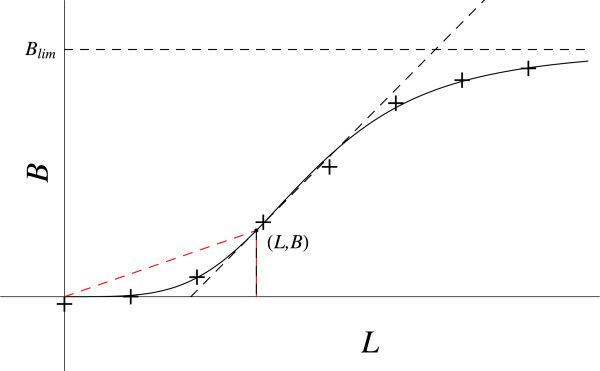
**Multiple ligand binding.** The red dashed line gives the coordinates slope (*B / L*). The black dashed line gives the slope at this point (*dB / dL*).

Consider an allosteric oligomer with multiple identical subunits, each with one binding site for the identical ligands, as in O_2_ binding to Hb [6]. The first stage of the data plot (Figure 6) is the binding of a ligand to one of the four unbound subunit sites on the Hb oligomer, each with a low affinity for O_2_ in the unbound T-state (the T-sites).

The three remaining unbound sites were assumed to transform (equivalently and simultaneously) into the R-state (the R-sites), due to the conformational change in Hb structure induced by the initial ligand binding to one of the T-sites [7]. These three, newly created, R-sites then exhibited an increased affinity for binding additional O_2_. The total number of binding sites on the oligomer was n_S_ = 4 = n_T_ + n_R_ = 1 + 3. Thus, n_T_ = 1 for whichever of the four, initially unbound, T-sites on the Hb oligomer bound the ligand in first-stage binding. Then n_R_ = 3 for the R-sites engaged in second-stage binding.

The experimentally observed sigmoidal curve of multi-site ligand binding, where *B* is the amount of bound sites and *L* is the ligand concentration, gives the classic Hill equation. At *L =* 0, then *B* = 0, and as *L* goes to infinity then *B* goes to B_lim._ The first derivative, (*dB* / *dL*), is always positive. The second derivative, *d*^2^*B* / *dL*^*2*^, is positive below the inflection point and negative above it. Thus, as previously

$$\frac{d^{2}B}{dL^{2}}=f\left[L,B,\left(\frac{\mathrm{dB}}{\mathrm{dL}}\right)\right]$$

where *d*^2^*B* / *dL*^*2*^ depends on *L, B,* and *dB* / *dL*, and *f* [] contains terms of the form *L*^p^ · *B*^q^ · (*dB* / *dL*)^r^ and the units on *f* [] must be (*B* / *L*^2^). This gives as before the two possibilities, (1 / *L*) and (1 / *B*) · (*dB* / *dL*). Regardless of the sign, neither alone can account for the changes in sign of the second derivative as it passes through the inflection point. The four linear combinations are then:

a. (1 /*L*) + (1 /*B*) · (*dB*/*dL*), always positive, so incorrect.

b. (− 1 /*L*) + (− 1 /*B*) · (*dB*/*dL*), always negative, so incorrect.

c. (1 /*L*) – (1 /*B*) · (*dB*/*dL*) = [(*dL* / *L*) – (*dB* / *B*)].

d. (− 1 /*L*) + (1 /*B*) · (*dB*/*dL*) = [− (*dL* / *L*) + (*dB* / *B*)].

Compare the coordinates slope, *B / L*, with the slope, *dB / dL*. Below the inflection point, [(*B / L*) < (*dB / dL*), and so (*dL / L*) < (*dB / B*). So case **c.** gives a negative RHS, incorrect. Then case **d**. gives the correct positive sign for the RHS, because the LHS is positive below the inflection point (tangent below the curve).

Introducing the integer coefficients, N = n_T_ + 1 and M = n_R_ + n_T_ + 1 = n_S_ + 1, for ligand binding to multiple sites on the same molecule, such as O_2_ binding to the four sites on a Hemoglobin molecule, gives for n_S_ total sites, with n_T_ = 1,

(6a) $$\frac{\frac{d^{2}B}{dL^{2}}}{\frac{\mathrm{dB}}{\mathrm{dL}}}\cdot \mathrm{dL}=\left(n_{T}+1\right)\cdot \frac{\mathrm{dB}}{B}-\left(n_{S}+1\right)\cdot \frac{\mathrm{dL}}{L}$$

Thus, m_1_ = n_S_ + 1 = 5, and m_2_ = n_T_ + 1 = 2. This is the same basic D.E. for the relationship of the variables as equation (5a), only with different values for m_1_ and m_2_, as well as different boundary conditions. After rearranging into the fractional change form and integrating,

(6b) $$\frac{\mathrm{dB}}{\mathrm{dL}}=C_{1}\cdot \frac{B^{2}}{L^{\left(n_{S}+1\right)}}$$

and

(6c) $$B=\frac{\left(1/C_{2}\right)\cdot L^{n_{S}}}{\frac{1}{n_{S}}\cdot \left(\frac{C_{1}}{C_{2}}\right)+L^{n_{S}}}$$

As *L* went to infinity, the limiting value went to

(6d) $$B_{\text{lim}}=\left(1/C_{2}\right)/\left(0+1\right)=1/C_{2}$$

Rewriting equation (6c) gave

(6e) $$B=\frac{B_{\text{lim}}\cdot L^{n_{S}}}{\left(C_{1}\cdot \frac{B_{\text{lim}}}{n_{S}}\right)+L^{n_{S}}}$$

Setting (C_1_ · B_lim_ / n_S_) = K_n_ illustrated its dependence on the number of binding sites present. In this case, K_n_ must have units of concentration to the power n_S_. Therefore, C_1_ has units of ${\left[\mathrm{mol}\;/\;L\right]}^{\left(n_{S}-1\right)}\cdot \min^{-1}={\left[\mathrm{mol}\;/\;L\right]}^{n_{R}}\cdot \min^{-1}$. In the single-site case, where now n_S_ = 1 and n_R_ = 0, this gave C_1_ = min = 1 / k. Thus, k = min^-1^ became the initial binding rate constant, the slope evaluated as *L* went to zero. For multiple-site binding, $1\;/\;C_{1}=k_{R}={\left[\mathrm{mol}\;/\;L\right]}^{{‒n}_{R}}\cdot \min^{-1}$. Using this gave (B_lim_ / n_S_ · k_R_) ≡ K_n_ and equation (6e) became

(6f) $$B=\frac{B_{\text{lim}}\cdot L^{n_{S}}}{K_{n}+L^{n_{S}}}$$

giving the Hill equation for multiple binding sites [6].

#### Enzyme catalysis

**Figure 7:**
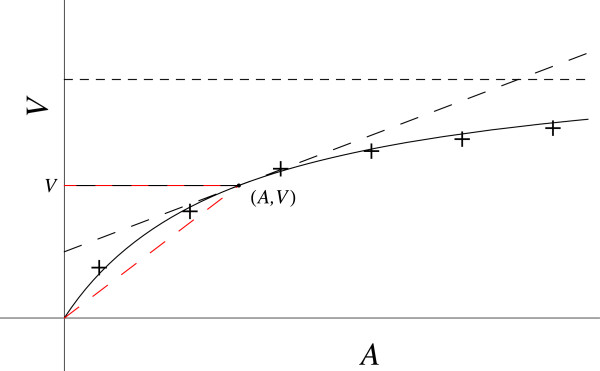
**Enzyme catalysis.** The red dashed line gives the coordinates slope (*v / A*). The black dashed line gives the slope at this point (*dv / dA*).

Numerous natural phenomena exhibit saturation behavior, including enzyme catalysis and ligand binding. For enzyme catalysis, let *v* be the catalytic reaction velocity and *A* the substrate concentration. Observation of the experimental data plot (Figure 7) for *v* versus *A* reveals the characteristic saturation behavior as *A* becomes very large.

The LHS of the D.E. is negative because the tangent to the data plot is above the curve, and the slope is positive. Both *d*^2^*v* / *dA*^2^ and *dv* / *dA* decrease as *A* increases. The Michaelis-Menten (M-M) equation for simple enzyme catalysis requires a second-order D.E., to be derived here, relating the variables (*A*, *v*, *dv / dA*) that is free of assumptions about mechanism and empirical constants. In general then,

$$\frac{d^{2}v}{dA^{2}}=f\left[v,A,\left(\frac{\mathrm{dv}}{\mathrm{dA}}\right)\right]$$

The units of the LHS are [*v / A*^2^]. As before there are eight distinct cases that satisfy the units requirement.

Cases **a.**, **c.**, **e.** and **g.** give a positive RHS, incorrect. Applying l’Hopital’s Rule to the RHS of the remaining cases for *A* goes to zero shows that only case **d.** will give a RHS = (*d*^2^*v* / *dA*^2^)_0_ = LHS.

Now introduce (as shown previously for the Hill equation) the integer coefficients N and M into the linear combination (**d.**) that define the roles of the binding sites, where N = n_T_ + 1

and

$$M=n_{R}+n_{T}+1$$

This gives

(7a) $$\frac{\left(\frac{d^{2}v}{dA^{2}}\right)\cdot \mathrm{dA}}{\left(\frac{\mathrm{dv}}{\mathrm{dA}}\right)}=\left(N\cdot \frac{\mathrm{dv}}{v}-M\cdot \frac{\mathrm{dA}}{A}\right)$$

This is the same basic D.E. as equations (5a) and (6a), with different values for m_1_ and m_2_, as well as different boundary conditions.

For M-M catalysis this simplifies to N = 1 + 1 = 2 = m_2_, and M = 0 + 1 + 1 = 2 = m_1_, because there is only one binding / catalytic site, see also [8].

(7b) $$\frac{\mathrm{dv}}{\mathrm{dA}}=C_{1}\cdot {\left(\frac{v}{A}\right)}^{2}$$

and

(7c) $$v=\frac{A}{C_{1}+C_{2}\cdot A}$$

The initial slope of the data plot yields C_1_ = 1 / k_bind_, which measures the binding interaction between a specific substrate and specific enzyme. As *A* increases, the limiting value of *v* is given by C_2_ = 1 / k_cat_, which measures the limiting rate of catalysis when the enzyme becomes saturated with substrate. Thus

(7d) $$v=\frac{1}{\frac{1}{A\cdot k_{\mathrm{bind}}}+\frac{1}{k_{\mathrm{cat}}}}$$

expresses the behavior of *v* solely in terms of the fundamental properties of the enzyme's catalytic function, k_bind_ and k_cat_, and its dependence on *A*. As expected, increasing any of these increases *v*.

The conventional formulation of equation (7d) is the classic M-M version:

(7e) $$v=\frac{V\cdot A}{K_{m}+A}$$

where V = k_cat_ · (total enzyme present). The Michaelis constant, K_m_, is actually a derived quantity, not a fundamental property of the enzyme's catalytic function, because it is defined by K_m_ = k_cat_ / k_bind_. It also differs from k_cat_ and k_bind_ because, in the presence of an inhibitor, neither k_cat_ nor k_bind_ is ever observed to increase, as expected, whereas in some circumstances K_m_ does increase (e.g., if the inhibitor acts only to decrease k_bind_). Thus, the D.E. leads uniquely to equation (7d), which identifies the importance of k_bind_ and clarifies the meaning of K_m_, see [8].

#### Logistic growth

**Figure 8:**
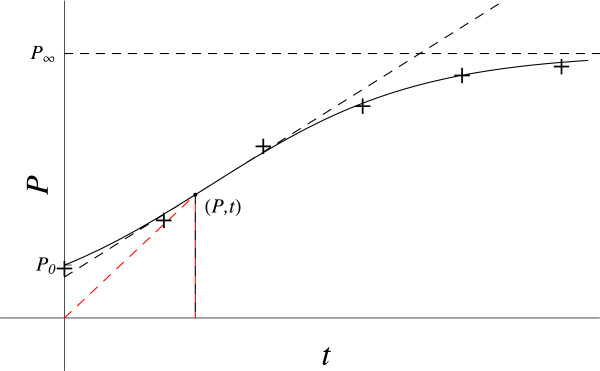
**Logistic growth.** The red dashed line gives the coordinates slope (*P / t*). The black dashed line gives the slope at this point (*dP / dt*).

Various equations have been developed to model general biological growth as well as population growth [9,10]. Typically, a first-order D.E. is postulated using the growth velocity, d*P* / d*t*. The approach developed here is the first to take up the idea presented by Ginzburg [11] that the second derivative of the population with respect to time (*d*^2^*P / dt*^2^), the growth acceleration, might be a useful variable for describing population growth. This common phenomenon (Figure 8) exhibits sigmoidal behavior differing mathematically from the sigmoidal Hill equation in having a finite value at zero time, the initial population P_0_.

Start with a general second-order D.E. relating the acceleration in population growth, *d*^2^*P / dt*^2^ to the relevant variables. Population growth depends only on the changes in the population, not on the time, *t,* and so the D.E. is autonomous. The acceleration at any time, *t*, depends on the population (*P*) available at *t*, on the remaining population growth available in the system, *P*_a_, and on the net production rate of new members (*dP / dt*)_*t*_ = [(population gain rate – loss rate) / time increment]_*t*_. So

(8a) $$\frac{d^{2}P}{dt^{2}}=f\left[P,\left(\mathrm{dP}/\mathrm{dt}\right),P_{a}\right]$$

To obtain the (1 / T^2^) term for the units on the RHS, *f* [] must include (*dP / dt*)^2^, therefore

(8b) $$\frac{d^{2}P}{dt^{2}}={\left(\frac{\mathrm{dP}}{\mathrm{dt}}\right)}^{2}\cdot g\left[P^{q},P_{a}^{r}\right]$$

Now, *P* / *T*^2^ = (*P*^2^/ *T*^2^) · (1 / *P*) is required, and so *g* [] has units of (1 / P) = P^-1^.

Consider then the value (-1) for q and r. The sign of *d*^2^*P / dt*^2^ changes from positive below the inflection point, to negative above it. The sign of (*dP / dt*)^2^ is always positive. Therefore, the sign of *g* [(*P*)^q^, (*P*_a_)^r^] must be positive below and negative above the inflection point. Consider linear combinations of (1 / *P*) — a growth promotion function that starts out large and decreases as population increases — and 1 / (*P*_a_) = 1 / (*P*_i_) that acts as a growth inhibitory function, which starts out small and increases in magnitude as population increases, because there are now fewer resources available for additional population growth. The question is how do they combine?

a. (1 / *P*) + (1 / *P*_i_), fails because it is always positive.

b. - (1 / *P*) – (1 / *P*_i_), fails because it is always negative.

c. - (1 / *P*) + (1 / *P*_i_), fails because when *t* is small, the magnitude of (1 / *P*) is greater than (1 / *P*_i_). Therefore, the RHS is negative, whereas the LHS, *d*^2^*P / dt*^2^, is positive (tangent below the curve).

d. (1 / *P*) – (1 / *P*_i_) is correct because it is positive below and negative above the inflection point where (1 / *P*) < (1 / *P*_i_), as required, giving

(8c) $$\frac{d^{2}P}{dt^{2}}={\left(\frac{\mathrm{dP}}{\mathrm{dt}}\right)}^{2}\cdot \left[\frac{1}{P}-\frac{1}{P_{i}}\right]$$

As *t* increases from zero, the first term dominates and reflects growth that is slowing as *P* increases towards the inflection point. After the inflection point, as *t* continues to increase, the limitations imposed by decreasing resources are reflected in the further decrease in (1 / *P*) and the increase in magnitude of (1 / *P*_i_), because *P*_i_ is getting smaller as *t* increases, giving a slowly increasing and negative RHS, as required. Assume *dP / dt = - dP*_a_*/ dt*, giving after integration,

(8d) $$\frac{\mathrm{dP}}{\mathrm{dt}}=C_{1}\cdot P\cdot P_{i}$$

Conservation requires that (*P*_a_ + *P*) = *P*_i_ + *P* = P_∞_, the limiting size of the population. Integrating gives

(8e) $$P=\frac{P_{\infty }}{1+C_{2}\cdot e^{-C_{1}\cdot t}}$$

the logistic function, where C_2_ = P_0,_ the starting population, and C_1_ = k, a measure of the growth rate in the presence of a limiting factor.

## Discussion

These results establish the basic principle that the mathematical properties of the experimental data plot specify a second-order D.E. describing that plot and the natural phenomenon that generates it. The derivation of the general D.E. involves only the variables. It is independent of assumptions about empirical constants and mechanisms. What makes this possible is the analytic power of the dimensional analysis restriction on the terms for each side of the D.E. equation.

The D.E. approach is simple, unifying, and revealing of the fundamental relationship among the variables. This is different from the relationships presented either by the algebraic function with its two empirical constants already specified, or by the first-order D.E. with its one specified empirical constant. The focus is on the dynamics of the change in acceleration (*d*^2^*y* / *dt*^2^) and its dependence on the velocity (*dy / dt*) — as modified by a particular linear combination of (1 / *t*) and (1 / *y*) · (*dy / dt*). It is the absence of empirical constants that allows this relationship to emerge.

Just two factors are invoked to specify the unique form of each second-order D.E. considered here, (1 / *t*) and (1 / *y*) · (*dy / dt*). The D.E.s revealed the non-linear dependence of *d*^2^*y / dt*^2^ on (1 / *t*) · (*dy / dt*) and (1 / *y*) · (*dy / dt*)^2^. The integrable fractional change form for each of these factors is, (*dt / t*) and (*dy / y*). A useful analytical concept is the relation between the coordinates slope (*y / t*) and the slope (*dy / dt*). It yields the relative magnitudes of the fractional changes in the variables for the data plot.

Each D.E. established the basic relationship among the variables describing each phenomenon. The appropriate empirical constants arise directly from the integrations and boundary conditions. Of particular interest was the ability of this analytical method to derive the D.E. for three related, but different, complex phenomena with different sigmoidal data plots — Standard Normal Distribution, Logistic Growth, and Hill Ligand Binding.

This analysis of the mathematical properties of the data plot for a specific natural phenomenon offers a new, simple, mechanism-independent method of deriving definitively the underlying D.E. for these natural phenomena. Often, different mechanisms can lead to the same function for describing a natural phenomenon. Even if such a mechanism derives the function describing the data plot, this does not establish definitively its validity. The Occam’s razor approach developed here avoids these problems.

The approach illustrates the analytical value of dimensional analysis in deriving differential equations that define the relationships among the variables, free of empirical constants. It develops the principle that each phenomenon gives unique mathematical properties to the data plot. The mathematical properties of the data plot are independent of mechanism, though not the converse. The defining relationships among the variables for each phenomenon reside in the experimental data plot. Reading the plot tells the phenomenon’s story.

## Competing interests

The author declares that he has no competing interests.
